# Supplementary material for: An open-source framework for large-scale, flexible evaluation of biomedical text mining systems
Source: J Biomed Discov Collab. 2008 Jan 29;3:1. doi: 10.1186/1747-5333-3-1 (PMC2276192; doi:10.1186/1747-5333-3-1)
Supplement: Additional file 1 — Evaluation platform source code. The additional file contains the software evaluation framework discussed in this paper. The most current release can be downloaded from SourceForge.net [1]. See the accompanying README file for instructions on installation and use. Also included in the distribution is a collection of UIMA wrappers for some commonly used BioNLP tools and annotated corpora. [file 1747-5333-3-1-S1.zip › bionlp_uima_repository-1.0.1/docs/api/src-html/edu/uchsc/ccp/uima/annotation/syntactic/CCPTypedDependency_Type.html]

```
001    
002    /* First created by JCasGen Tue Aug 28 16:44:10 MDT 2007 */
003    package edu.uchsc.ccp.uima.annotation.syntactic;
004    
005    import org.apache.uima.cas.Feature;
006    import org.apache.uima.cas.FeatureStructure;
007    import org.apache.uima.cas.Type;
008    import org.apache.uima.cas.impl.CASImpl;
009    import org.apache.uima.cas.impl.FSGenerator;
010    import org.apache.uima.cas.impl.FeatureImpl;
011    import org.apache.uima.cas.impl.TypeImpl;
012    import org.apache.uima.jcas.JCas;
013    import org.apache.uima.jcas.JCasRegistry;
014    import org.apache.uima.jcas.cas.TOP_Type;
015    
016    /** This typed dependency structure was created to hold typed dependency information generated by the Stanford Parser.
017     * Updated by JCasGen Tue Aug 28 16:44:10 MDT 2007
018     * @generated */
019    public class CCPTypedDependency_Type extends TOP_Type {
020      /** @generated */
021      protected FSGenerator getFSGenerator() {return fsGenerator;}
022      /** @generated */
023      private final FSGenerator fsGenerator = 
024        new FSGenerator() {
025          public FeatureStructure createFS(int addr, CASImpl cas) {
026                             if (CCPTypedDependency_Type.this.useExistingInstance) {
027                               // Return eq fs instance if already created
028                         FeatureStructure fs = CCPTypedDependency_Type.this.jcas.getJfsFromCaddr(addr);
029                         if (null == fs) {
030                           fs = new CCPTypedDependency(addr, CCPTypedDependency_Type.this);
031                               CCPTypedDependency_Type.this.jcas.putJfsFromCaddr(addr, fs);
032                               return fs;
033                         }
034                         return fs;
035            } else return new CCPTypedDependency(addr, CCPTypedDependency_Type.this);
036              }
037        };
038      /** @generated */
039      public final static int typeIndexID = CCPTypedDependency.typeIndexID;
040      /** @generated 
041         @modifiable */
042      public final static boolean featOkTst = JCasRegistry.getFeatOkTst("edu.uchsc.ccp.uima.annotation.syntactic.CCPTypedDependency");
043     
044      /** @generated */
045      final Feature casFeat_governorTokenNum;
046      /** @generated */
047      final int     casFeatCode_governorTokenNum;
048      /** @generated */ 
049      public int getGovernorTokenNum(int addr) {
050            if (featOkTst && casFeat_governorTokenNum == null)
051          jcas.throwFeatMissing("governorTokenNum", "edu.uchsc.ccp.uima.annotation.syntactic.CCPTypedDependency");
052        return ll_cas.ll_getIntValue(addr, casFeatCode_governorTokenNum);
053      }
054      /** @generated */    
055      public void setGovernorTokenNum(int addr, int v) {
056            if (featOkTst && casFeat_governorTokenNum == null)
057          jcas.throwFeatMissing("governorTokenNum", "edu.uchsc.ccp.uima.annotation.syntactic.CCPTypedDependency");
058        ll_cas.ll_setIntValue(addr, casFeatCode_governorTokenNum, v);}
059        
060      
061     
062      /** @generated */
063      final Feature casFeat_dependentTokenNum;
064      /** @generated */
065      final int     casFeatCode_dependentTokenNum;
066      /** @generated */ 
067      public int getDependentTokenNum(int addr) {
068            if (featOkTst && casFeat_dependentTokenNum == null)
069          jcas.throwFeatMissing("dependentTokenNum", "edu.uchsc.ccp.uima.annotation.syntactic.CCPTypedDependency");
070        return ll_cas.ll_getIntValue(addr, casFeatCode_dependentTokenNum);
071      }
072      /** @generated */    
073      public void setDependentTokenNum(int addr, int v) {
074            if (featOkTst && casFeat_dependentTokenNum == null)
075          jcas.throwFeatMissing("dependentTokenNum", "edu.uchsc.ccp.uima.annotation.syntactic.CCPTypedDependency");
076        ll_cas.ll_setIntValue(addr, casFeatCode_dependentTokenNum, v);}
077        
078      
079     
080      /** @generated */
081      final Feature casFeat_relation;
082      /** @generated */
083      final int     casFeatCode_relation;
084      /** @generated */ 
085      public String getRelation(int addr) {
086            if (featOkTst && casFeat_relation == null)
087          jcas.throwFeatMissing("relation", "edu.uchsc.ccp.uima.annotation.syntactic.CCPTypedDependency");
088        return ll_cas.ll_getStringValue(addr, casFeatCode_relation);
089      }
090      /** @generated */    
091      public void setRelation(int addr, String v) {
092            if (featOkTst && casFeat_relation == null)
093          jcas.throwFeatMissing("relation", "edu.uchsc.ccp.uima.annotation.syntactic.CCPTypedDependency");
094        ll_cas.ll_setStringValue(addr, casFeatCode_relation, v);}
095        
096      
097    
098    
099    
100      /** initialize variables to correspond with Cas Type and Features
101            * @generated */
102      public CCPTypedDependency_Type(JCas jcas, Type casType) {
103        super(jcas, casType);
104        casImpl.getFSClassRegistry().addGeneratorForType((TypeImpl)this.casType, getFSGenerator());
105    
106     
107        casFeat_governorTokenNum = jcas.getRequiredFeatureDE(casType, "governorTokenNum", "uima.cas.Integer", featOkTst);
108        casFeatCode_governorTokenNum  = (null == casFeat_governorTokenNum) ? JCas.INVALID_FEATURE_CODE : ((FeatureImpl)casFeat_governorTokenNum).getCode();
109    
110     
111        casFeat_dependentTokenNum = jcas.getRequiredFeatureDE(casType, "dependentTokenNum", "uima.cas.Integer", featOkTst);
112        casFeatCode_dependentTokenNum  = (null == casFeat_dependentTokenNum) ? JCas.INVALID_FEATURE_CODE : ((FeatureImpl)casFeat_dependentTokenNum).getCode();
113    
114     
115        casFeat_relation = jcas.getRequiredFeatureDE(casType, "relation", "uima.cas.String", featOkTst);
116        casFeatCode_relation  = (null == casFeat_relation) ? JCas.INVALID_FEATURE_CODE : ((FeatureImpl)casFeat_relation).getCode();
117    
118      }
119    }
120    
121    
122    
123
```
